# ClC-3 mediates angiotensin II-induced endothelial dysfunction by inhibiting Akt-Hsp90-eNOS signaling pathway

**DOI:** 10.3389/fphar.2026.1795707

**Published:** 2026-03-27

**Authors:** Lei Li, Yu-quan Lin, Yu-sheng Peng, Huan-hao Chen, Xiao-jun Wu, Guozheng Liang, Yan-hua Du

**Affiliations:** 1 Department of Pharmacology, Cardiac and Cerebral Vascular Research Center, Zhongshan School of Medicine, Sun Yat-Sen University, Guangzhou, China; 2 Department of Pharmacy, The Second Affiliated Hospital of Chongqing Medical University, Chongqing, China; 3 Department of Cardiovascular Surgery of the First Affiliated Hospital and Institute for Cardiovascular Science, Suzhou Medical College, Soochow University, Suzhou, China

**Keywords:** angiotenin II, ClC-3, endothelial dysfunction, hypertension, nitric oxide

## Abstract

**Background:**

Endothelial dysfunction is a major driver for hypertension and diabetes. Chloride (Cl^−^) is the most abundant anion in vascular endothelial cells (ECs). However, the role of chloride channel or chloride/proton exchanger ClC-3 in the vascular endothelium and its relationship with endothelium-dependent relaxation remains unclear.

**Methods:**

This study aims to explore the role and mechanism of ClC-3 in endothelial dysfunction during hypertension, by using primary cultured human umbilical vein endothelial cells (HUVECs) and ClC-3 knockout mice.

**Results:**

We found that AngiotensinII (AngII) treatment significantly upregulated ClC-3 expression and reduced NO levels, while ClC-3 interference increased eNOS phosphorylation. Furthermore, in AngII-infused hypertensive mouse models, ClC-3 knockout significantly increased the phosphorylation level of eNOS and improved vascular relaxation function. *In vitro* data shows that ClC-3 interacted with eNOS through its oxygenase domain in HUVECs. Moreover, lack of ClC-3 promoted eNOS-Hsp90-Akt complex formation and eNOS disassociation from caveolin-1, and upregulation the phosphorylation of Akt.

**Discussion:**

Our findings suggest that ClC-3 may serve as an important target for hypertension-related endothelial dysfunction, potentially providing new strategies and interventions for the treatment of hypertension and its complications.

## Introduction

Hypertension is a multifactorial disorder influenced by both genetic and environmental factors such as high-salt intake, and remains a major risk factor for cardiovascular diseases as well as diabetes worldwide (Elijovich et al., 2016; Koenen et al., 2021; Adachi et al., 2025). A significant pathological alteration underlying hypertension is endothelial dysfunction, characterized by reduced bioavailability of nitric oxide (NO) and subsequent impairment of endothelium-dependent relaxation (Ma et al., 2023). Endothelial cells are pivotal regulators of the vascular microenvironment, and their production of NO is crucial for maintaining vascular relaxation and overall cardiovascular health (Augustin and Koh, 2024; Pasut et al., 2025). The synthesis of NO is primarily dependent on the activation of endothelial nitric oxide synthase (eNOS), a process tightly regulated by various transcriptional and post-translational modifications. In the hypertensive pathophysiological process, eNOS activity is often inhibited, leading to reduced NO production and resulting in endothelial dysfunction (Huang et al., 1995; Brandes, 2014).

While extensive research has focused on cations such as Ca^2+^ and Na^+^ in cardiovascular regulation (Chen et al., 2022; Pandey et al., 2023), the role of chloride (Cl^−^), the principal intracellular and extracellular anion, has long been underestimated. Increasing evidence indicates that serum Cl^−^ levels are tightly associated with cardiovascular risk, and hypochloremia is significantly correlated with adverse outcomes in hypertensive patients (Goto and Kitazono, 2022; Hou et al., 2023; Ferreira et al., 2017). This highlights Cl^−^ as a critical, yet underappreciated, component of vascular homeostasis. Despite the known link between Cl^−^ dysregulation and adverse hypertensive outcomes (Mata-Daboin et al., 2025), the specific molecular mechanisms by which chloride related pathway may modulate eNOS activity and drive endothelial dysfunction remain largely unknown.

ClC-3, a member of the voltage-dependent chloride channel family or chloride/proton exchanger, is expressed in cell membranes, organelles, and cell nuclei, and regulates various physiological and pathological functions of cells, including cell volume regulation, membrane potential, acid-base balance, and processes such as proliferation, differentiation, and apoptosis (Raut et al., 2024; Duan, 2011; Wan et al., 2024). Previous study has shown that ClC-3 is involved in hypertension-related cerebrovascular remodeling in vascular smooth muscle cells (Zheng et al., 2013). Additionally, early research found that ClC-3 is expressed in endothelial cells and may affect endothelial cell function by regulating inflammatory factors formation and reactive oxygen species (ROS) levels (Liang et al., 2018; Liu et al., 2013; Yang et al., 2012). This provides a possibility for ClC-3 as a new target for hypertension treatment, but whether ClC-3 participates in the regulation of vasodilation and the endothelial specific mechanisms still need further exploration.

Therefore, this study aims to investigate the role of ClC-3 in endothelial dysfunction in hypertension and its mechanisms. We will use primary cultured human umbilical vein endothelial cells (HUVECs) and ClC-3 knockout mouse models to observe the effects of altering ClC-3 expression levels on endothelial dependent relaxation. Additionally, we will focus on analyzing the interactions between ClC-3, eNOS, and their signaling pathways to clarify whether ClC-3 is a regulatory factor for endothelial dysfunction in hypertension. Through these studies, we hope to provide new experimental evidence for the prevention and treatment of hypertension and its related complications, further promoting research progress in cardiovascular diseases.

## Materials and methods

### Cell isolation and culture

HUVECs were isolated and cultured as described previously (Liang et al., 2018). The experiments were approved by the medical research ethics committee of Sun Yat-Sen University and conducted according to the principles expressed in the Declaration of Helsinki. Informed consents were obtained from all subjects. In brief, HUVECs were harvested from the umbilical vein digested by 0.125% trypsin with 0.01% EGTA. Then the cells were cultured in M199 medium containing 20% fetal calf serum, 100 U/mL penicillin, 100 U/mL streptomycin, 25 U/mL heparin, 2 mmol/L L-glutamine, and 5 ng/mL p-ECGF at 37 °C, 5% CO_2_ humidified atmosphere. Cells were used between passages 4 and 8 in this study.

### ClC-3 shRNA adenoviral infection

Ad-ClC-3 shRNA was designed and produced by Sunbio Medical Biotechnology (Shanghai, CN). The sequence of Ad-ClC-3 shRNA is 5′- CCA​CGA​CTG​GTT​TAT​CTT​T-3’. Briefly, cultured HUVECs at 50% confluence were infected with adenovirus encoding ClC-3 shRNA for 6 h in M199 culture medium containing 2% FBS, then washed and incubated in fresh normal M199 medium for an additional 48 h before experiment.

### Animal model

ClC-3^−/−^ mice and its corresponding ClC-3^+/+^ controls of 129/SvJ-C57BL/6 background, as kind gifts of Dr. Duan (University of Nevada, School of Medicine, Reno, Nevada, USA), were bred in house. Genotype was screened by polymerase chain reaction (PCR) analysis of DNA isolated from tail clips using ClC-3 gene specific primers as previously (Yang et al., 2012). All animals were maintained with standard food and water in pathogen-free facilities with a 12-h light/dark cycle. All experimental procedures were performed in accordance with the policies of the Sun Yat-Sen University Animal Care and Use Committee and conformed to the “Guide for the Care and Use of Laboratory Animals” of the National Institute of Health in China.

AngII-induced hypertensive mice were operated according to manufacturer’s instruction. Briefly, AngII (1.5 mg/kg/d) or saline was infused into Alzet mini-osmotic pumps (Model 1002, Alzet Durect Corp, Cupertino, CA). Mice (18–22 g, male) were anesthetized intraperitoneally with 40 mg/kg pentobarbital sodium. Then the infused pumps were implanted subcutaneously between the scapulae. Systolic blood pressure (SBP) was measured in conscious mice by noninvasive, tail-cuff plethysmography (Powerlab 4/30, AD Instruments, AU) as previously described. The blood pressure measurements were taken 5 times consecutively for each mouse. The averaged data represents the blood pressure at that time point. The tissues were collected 2 weeks after AngII implantation, after the mice were sacrificed by overdose of pentobarbital intraperitoneally.

### Measurement of vasorelaxation

Vasorelaxation was evaluated by organ chamber (DMT 620M, Winnipeg, CA), according to the manufacturer’s protocol. Briefly, mice were anesthetized with pentobarbital sodium (40 mg/kg). Aortas were removed immediately and separated carefully. After preparation of the vessels, endothelium-dependent relaxation was assessed with cumulative doses of acetylcholine (ACh, 10^–9^ to 10^–5^ mol/L) after precontraction with phenylephrine (PE, 1 μmol/L). In a separate group, NO-mediated relaxation was verified by NOS inhibitor L-NAME (10 μmol/L) preincubated for 15 min before Ach administration. In complementary experiments, endothelium-independent responses to sodium nitroprusside (SNP, 10^–11^ to 10^–5^ mol/L) were performed. The relaxation responses were expressed as a percent relaxation of PE-induced contraction.

### Western blot

Western blot was performed as previously described (Yang et al., 2012). Briefly, HUVECs were lysed with RIPA lysis buffer (20 mM Tris, pH 7.5, 150 mM NaCl, 1% Triton X-100, sodium pyrophosphate, β-glycerophosphate, EDTA, Na3VO4, leupeptin) containing protease inhibitor cocktail (Merck, Germany) and phosphatase inhibitor (PhosSTOP, Roche, USA). Protein was separated with 8% SDS-PAGE and transformed to PVDF membrane. After blocked with 5% milk at room temperature for 1 h, the membrane was incubated with primary antibodies against ClC-3 (1:200, Alomone), against total eNOS (1:1000, BD Transduction Laboratories), against p-eNOS (Ser-1177, 1:1000, Cell Signaling Technology), against Akt and p-Akt (1:1000, Cell Signaling Technology), or against AMPK and p-AMPK (1:1000, Cell Signaling Technology) at 4 °C overnight, and then incubated for 1 h with secondary antibody at room temperature. Bands were detected with Pierce ECL western blotting substrate and quantified with the ImageJ software.

### Co-immunoprecipitation

Co-immunoprecipitation was performed according to the protocol previously described (Yang et al., 2012). Briefly, cell lysates containing 500–600 μg protein were incubated with antibodies overnight at 4 °C. Then 15 μL protein A/G PLUS-Agrose was added followed by incubating in shaker for 4 h at 4 °C. After centrifugation at 2,500 rpm at 4 °C for 15 min, the supernatant was discarded, and the pallet was washed twice with ice-cold PBS, then boiled in protein SDS sample buffer. Samples were resolved on 8% SDS-PAGE gels and transferred to PVDF membranes. The bound proteins were determined by immunoblotting with the indicated antibodies.

### Measurement of NO in HUVECs

HUVECs were incubated with or without 10^–6^ mol/L AngII. Some of cells were pretreated with Cl^−^ channel blockers before AngII administration. The stimulated NO production was confirmed by laser confocal fluorescent microscopy using a specific dye, 4-Amino-5-Methylamino-2′,7′-Difluorofluorescein Diacetate (DAF-FM DA, Molecular Probes, USA). Micrographs were taken by the confocal microscope and fluorescent intensity was analyzed by Image J.

NO production in HUVECs was also examined by quantifying its degradation products using Nitrate/Nitrite Assay Kit according to the manufacturer’s instruction. Each sample was performed in triplicate.

### Statistical analysis

All statistical analyses were performed using GraphPad Prism 5 (GraphPad software, La Jolla, CA). All data were expressed as mean ± SEM. N represented the number of independent experiments on different batches of cells or different mice. An unpaired 2-tailed Student t test was used to detect significant differences between 2 groups. One-way or two-way ANOVA followed by Bonferroni multiple comparison test was used to compare differences if there are more than 2 groups. Values of P < 0.05 were considered statistically significant.

## Results

### ClC-3 deficiency improves endothelium-dependent vasodilation in AngII-induced hypertensive mice

**FIGURE 1:**
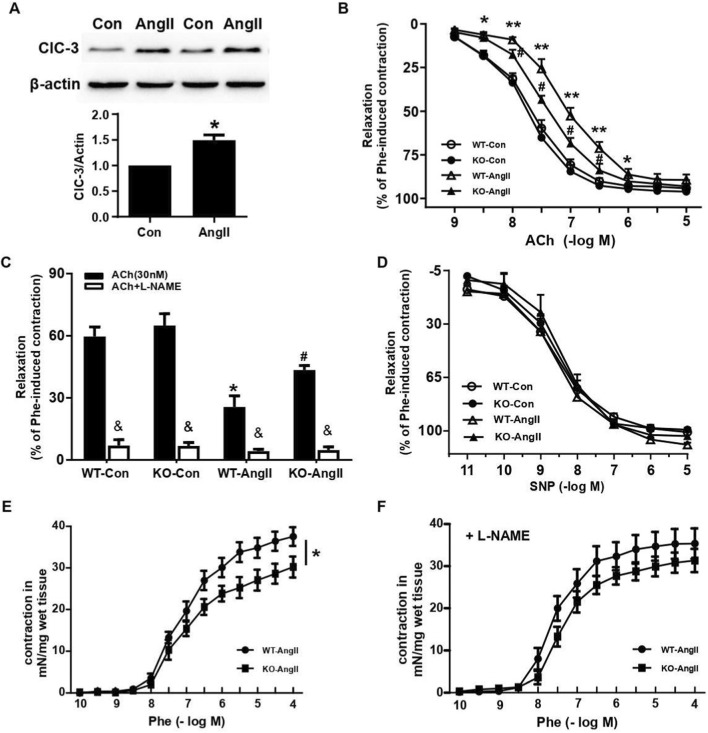
Effects of ClC-3 on Ach-mediated relaxation in aortas from AngII-induced hypertensive mice. **(A)** The expression of ClC-3 was increased in HUVECs treated with AngII (1 μmol/L) for 24 h (n = 6, *P < 0.05 vs. Con). **(B)** Knockout of ClC-3 improved endothelial dysfunction induced by AngII. Endothelial function was assayed by Ach-induced endothelium-dependent relaxation in aortas from WT and ClC-3 KO mice with or without AngII infusion (n = 10, *P < 0.05, **P < 0.01 vs. WT-Con; ^#^P < 0.05 vs. WT-AngII). **(C)** Ach-induced vasodilation was eliminated by pretreatment with 10 μmol/L NO synthase inhibitor L-NAME (n = 6, *P < 0.01 vs. WT-Con; ^#^P < 0.05 vs. WT-AngII; ^&^P < 0.01 vs. Ach). **(D)** There was no difference in SNP-induced endothelium-independent relaxation among groups (n = 7). **(E,F)** The vasoconstriction response to phenylephrine in aortas from AngII-infused WT and ClC-3 KO mice with or without L-NAME pretreatment. The difference of aortic contraction to phenylephrine between WT and KO groups was attenuated by pretreatment with L-NAME (n = 8–10, *P < 0.05 KO-AngII vs. WT-AngII).

As shown in Figure 1A, the expression of ClC-3 was significantly increased in Ang II (1 μmol/L)-treated HUVECs. In order to evaluate the role of ClC-3 in endothelial function during hypertension, ClC-3 knockout mice were implanted with AngII-infused pumps for 2 weeks, and vascular relaxation function was investigated. In normal condition, no differences were found between the endothelium-dependent vasodilation responses in the aortas from wild type (WT) and ClC-3 knockout (KO) mice. However, the endothelium-dependent relaxations to ACh (from the concentration of 3 × 10^−9^ to 10^–6^ mol/L) were impaired in AngII-infused WT hypertensive mice. Importantly, this impairment was significantly improved by ClC-3 knockout (Figure 1B), although the blood pressure was elevated in both WT and ClC-3 KO mice to similar levels (Supplementary Figure S1). Furthermore, these relaxation responses to ACh were abrogated by pre-incubating with L-nitro-arginine methylester (L-NAME) (1 μmol/L), a NO synthase inhibitor (Figure 1C). Conversely, endothelium-independent relaxations to the NO donor SNP were identical in the 4 groups, suggesting no difference in vascular smooth muscle responses to exogenous NO (Figure 1D). In addition, the concentration-dependent contractile response induced by phenylephrine in intact aortic rings was weaker in hypertensive ClC-3 deficient mice than that in WT mice (Figure 1E). However, this difference was abrogated by pretreatment with L-NAME, implying a higher basic NO generation in ClC-3 knockout mice (Figure 1F). Together, these observations suggest that ClC-3 was involved in AngII-induced endothelial dysfunction. Deficiency of ClC-3 significantly preserved NO-mediated relaxation.

### ClC-3 deficiency restores NO levels and eNOS phosphorylation in AngII-treated HUVECs and hypertensive arteries

**FIGURE 2:**
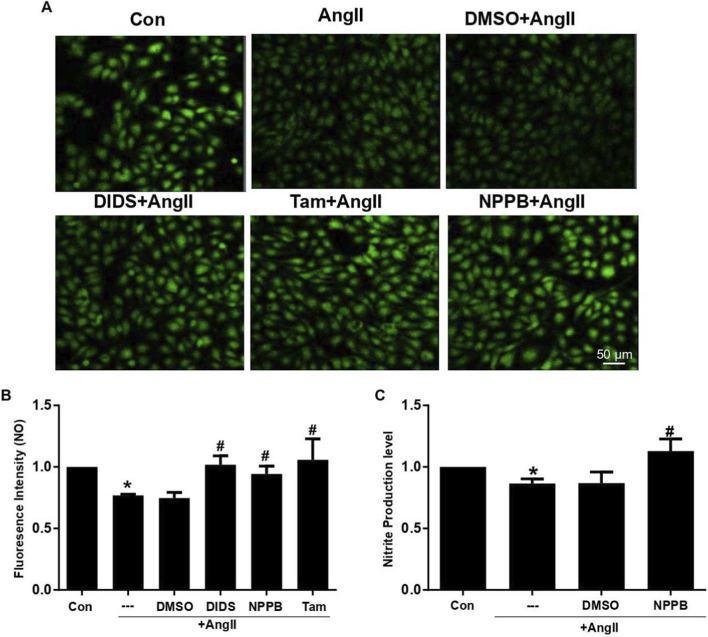
Cl^−^ channel blockers restored NO production in HUVECs. **(A,B)** HUVECs were pretreated with Cl^−^ channel blocker (200 μM DIDS, 20 μM Tamoxifen or 100 μM NPPB) for 30 min before being subjected to 24 h AngII. NO production was measured by DAF-FM DA. Representative images were taken by the confocal microscope (A, 200X, bar length: 50 μm). The amount of NO analyzed by measuring the fluorescence intensity was shown (B, *P < 0.01 vs. control; ^#^p < 0.05 vs. AngII). **(C)** NO production in HUVECs was examined by Nitrate/Nitrite Assay Kit (*P < 0.01 vs. control; ^#^p < 0.05 vs. AngII). n = 5.

To explore the mechanism by which ClC-3 deficiency preserves endothelium-dependent relaxation, we next examined endothelial NO production and eNOS phosphorylation in AngII-treated HUVECs and mouse thoracic arteries. Fluorescence analysis of DAF and nitrate/nitrite assays revealed that AngII (10 μM, 24 h) significantly decreased NO levels in HUVECs. Preincubation with chloride channel blockers (DIDS, NPPB, or Tamoxifen) restored NO production, indicating that Cl^−^ flux contributes to AngII-induced NO suppression (Figures 2A–C).

**FIGURE 3:**
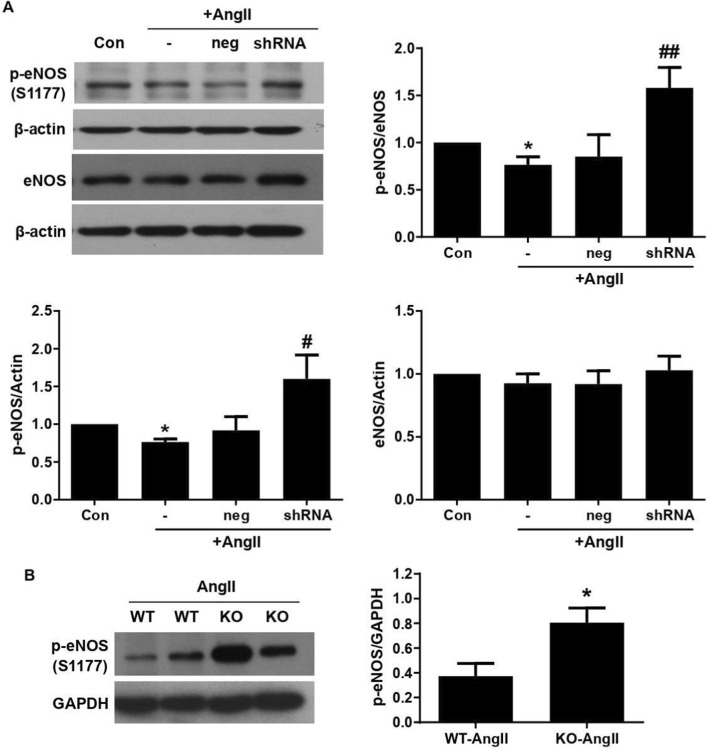
Effect of ClC-3 on the expression and phosphorylation of eNOS at serine 1177. **(A)** HUVECs were treated with the 100 MOI adenovius ClC-3 shRNA for 48 h, then 1 μM AngII was added for an additional 24 h. AngII decreased the eNOS phosphorylation at serine 1177. This effect was reversed by ClC-3 knockdown (*P < 0.05 vs. control; ^#^P < 0.05 and ^##^P < 0.01 vs. AngII, n = 5). Values were normalized to control. **(B)** Western blot analysis of eNOS phosphorylation at serine 1177 in thoracic aortas from AngII-infused wild-type (WT) and ClC-3 knockout (KO) mice. Representative images showed that ClC-3 KO increased eNOS phosphorylation in hypertensive mouse aorta (*P < 0.05 vs. WT-AngII, n = 5). Results are mean ± SEM.

Consistent with NO measurements, ClC-3 knockdown in HUVECs using shRNA significantly increased eNOS phosphorylation at Ser1177 under basal conditions (Supplementary Figure S2). Importantly, in AngII-treated cells, phospho-eNOS expression was markedly reduced, whereas ClC-3 knockdown reversed this decline in phospho-eNOS levels (Figure 3A). The transfection efficiency of ClC-3 shRNA was shown in Supplementary Figure S3.

*In vivo*, ClC-3 deficiency had no significant effect on total eNOS expression in thoracic arteries during hypertension (Supplementary Figure S4), but markedly increased eNOS phosphorylation (Figure 3B). These findings indicate that ClC-3 deficiency enhances NO bioavailability primarily through promoting eNOS activation rather than increasing eNOS expression. These results are consistent with the preserving NO-mediated endothelium-dependent relaxation in ClC-3 knockout mice (Figure 1E).

Together, these data suggest that loss of ClC-3 prevents AngII-induced NO decline by restoring eNOS phosphorylation, providing a mechanistic explanation for the improved endothelium-dependent relaxation observed in ClC-3-deficient hypertensive mice.

### Knockdown of ClC-3 enhanced the phosphorylation of Akt but not AMPK in HUVECs

**FIGURE 4:**
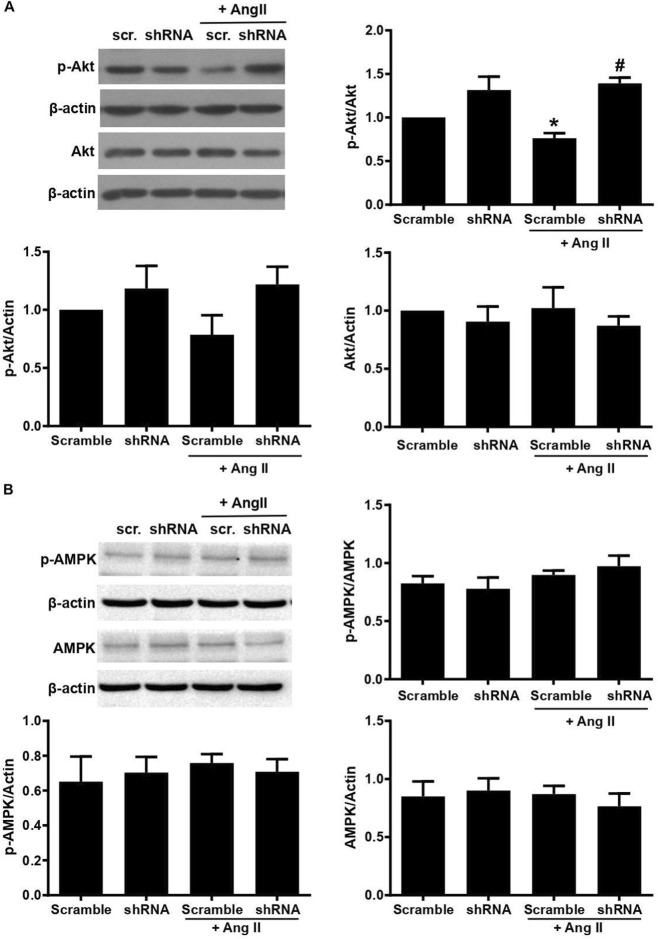
Effect of ClC-3 on the expression and phosphorylation of Akt and AMPK in AngII-treated HUVECs. **(A)** AngII decreased Akt phosphorylation, which was reversed by ClC-3 knockdown (*P < 0.05 vs. Scramble RNA control; ^#^P < 0.01 vs. Scr.RNA + AngII, n = 5). Values were normalized to the control. **(B)** There was no significant difference among groups referring to the expression of p-AMPK and AMPK. n = 4. Results are mean ± SEM.

Because Akt and AMPK are the two major kinases that promote eNOS phosphorylation, we determined the expression of these proteins in HUVECs. Our results showed that total Akt expression was similar among groups, as indicated by the ratio of total Akt to actin. The ratio of phosphorylated Akt to total Akt at the serine 473 residue (Akt-S473) was significantly decreased in AngII-treated HUVECs compared with control; however, this ratio of phosphorylated Akt to total Akt was preserved in ClC-3 shRNA transfected mice (Figure 4A). Interestingly, we observed no difference in the expression of both AMPK total protein and phosphorylation among the groups (Figure 4B). These results suggested that the increased eNOS phosphorylation in AngII-treated ClC-3 knockdown cells may be related to the elevation of Akt activation but not AMPK.

### Knockdown of ClC-3 increased the interaction of eNOS with Akt and Hsp90 in HUVECs

**FIGURE 5:**
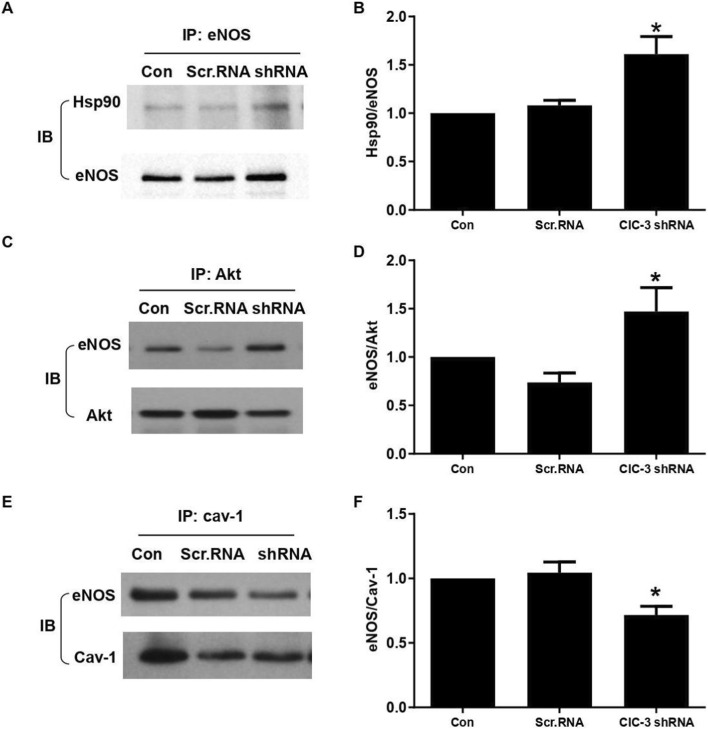
Effect of ClC-3 on the interaction of eNOS with Akt, Hsp90 and caveolin-1 in HUVECs. **(A,B)** After immunoprecipitation were probed with eNOS, western blot for Hsp90 was performed. Representative images showed that ClC-3 knockdown promoted Hsp90 association with eNOS, normalized to the internal standard eNOS. **(C,D)** Cell lysates were immunoprecipitated with Akt, then detected with eNOS antibody. Representative images showed that ClC-3 knockdown promoted the association of Akt with eNOS, normalized to the internal standard Akt. **(E,F)** HUVECs lysates were immunoprecipitated with caveolin-1 (cav-1) antibody and then the immunoprecipitates were probed with eNOS antibody by western blot. ClC-3 knockdown reduced the association of caveolin-1 with eNOS, normalized to the internal standard cav-1. Results are mean ± SEM, *P < 05 vs. Scr.RNA, n = 4. All the western blot data were normalized with control.

It was generally known that eNOS phosphorylation by Akt is highly dependent on its interaction with Hsp90. Therefore, we next investigated whether ClC-3 has an effect on the Hsp90/eNOS/Akt complex formation. As shown in Figure 5, the co-immunoprecipitation assay revealed that knockdown of ClC-3 by shRNA significantly increased the interaction of Hsp90 with eNOS, compared with control (Figures 5A,B). Simultaneously, the association between Akt and eNOS was also markedly increased by knockdown of ClC-3 (Figures 5C,D).

### Knockdown of ClC-3 promoted the dissociation of eNOS from caveolin-1

By binding with caveolin-1, eNOS is basically maintained in an inactive state in the membrane of endothelial cells. It is only on the release of eNOS from caveolin-1 that the enzyme can be fully activated. Therefore, we next examined whether ClC-3 influences the interaction of eNOS with caveolin-1. Our results showed that compared with control, knockdown of ClC-3 remarkably decreased the binding of eNOS to caveolin-1, reflected by the inhibited co-immunoprecipitation (Figures 5E,F). These data suggested that deficiency of ClC-3 promoted the release of eNOS from caveolin-1, which may contribute to the eNOS activation by Hsp90 and Akt.

### ClC-3 interacted with eNOS through its oxygenase domain

**FIGURE 6:**
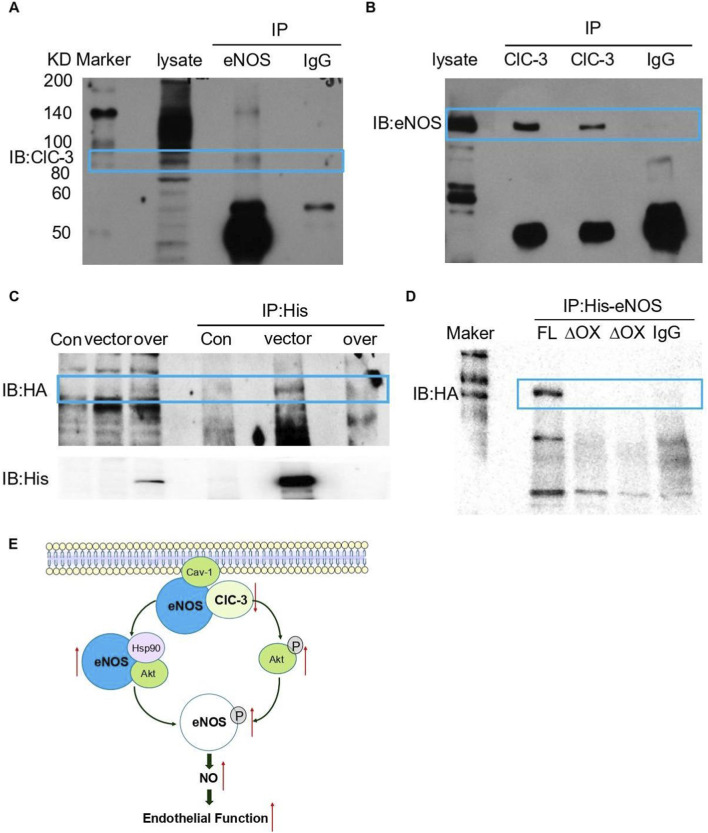
ClC-3 interacted with eNOS through its oxygenase domain. **(A,B)** The interaction of endogenous ClC-3 and endogenous eNOS in HUVECs. Cell lysates were immunoprecipitated with eNOS or ClC-3 antibody and then probed with ClC-3 or eNOS antibody, respectively. Normal IgG was used as negative control. **(C)** The interaction of exogenous ClC-3 and exogenous eNOS in COS-7 cells. Cells were co-transfected with exogenous Adenovirus-HA-ClC-3 and His-eNOS cDNA. The cell lysates were immunoprecipitated with anti-His antibody and then detected with anti-HA antibody by western blot. Normal IgG was used as negative control. The respective images showed that ClC-3 had an interaction with eNOS, n = 4. **(D)** COS-7 cells were co-transfected with HA-tagged ClC-3 and His-eNOS cDNA, including eNOS (full length, FL) and eNOS-ΔOX plasmids, respectively. The cell lysates were immunoprecipitated with anti-His antibody and then detected with anti-HA antibody by western blot. Normal IgG was used as negative control. n = 4. The interaction between ClC-3 protein and eNOS was absent in cells transfected with eNOS-ΔOX plasmid. **(E)** Schematic representation showing that ClC-3 deficiency promotes the formation of the Akt–Hsp90–eNOS signaling complex and upregulates Akt phosphorylation, which in turn increases eNOS phosphorylation and NO production, thereby restoring endothelium-dependent vasodilation.

To investigate the mechanism how ClC-3 regulates Akt and Hsp90 association with eNOS. In the co-immunoprecipitation (CO-IP) assay experiment, we found that endogenous ClC-3 has a direct interaction with eNOS in HUVECs (Figures 6A,B). To verify this result, we co-transfected eNOS and ClC-3 plasmids or viruses into COS-7 cells, which do not endogenously express eNOS. The results confirmed that this protein interaction was still detectable under conditions of exogenous overexpression (Figure 6C). Collectively, these findings demonstrate that there is an direct and specific interaction between these two proteins.

To determine which part of the eNOS protein was responsible for the interaction with ClC-3, truncated mutant of eNOS oxygenase domain (eNOS ΔOX) were generated, and the interaction between ClC-3 and the truncated eNOS was examined using CO-IP. The result showed that truncation of the oxygenase domain (1–511 aa) in the eNOS protein abolished its association with ClC-3, indicating eNOS might interact with ClC-3 through its oxygenase region (Figure 6D).

## Discussion

Chloride and chloride channels have emerged as pivotal regulators of vascular tone and cellular signaling beyond simple ion transport (Garrud et al., 2024; Sun et al., 2025; Klemens et al., 2021). Our study identifies the volume-sensitive chloride channel ClC-3 as a previously underappreciated negative regulator of eNOS activity in an AngII-driven hypertension model. We found that genetic ablation of ClC-3 restored acetylcholine-mediated vasorelaxation and increased eNOS phosphorylation at Ser1177, thereby enhancing NO production and improving endothelial function. This finding is supported by our previous work, which showed that ClC-3 promotes AngII-induced ROS production (Liang et al., 2018). Therefore, deficiency of ClC-3 may both increase NO synthesis and reduce NO scavenging. This is particularly significant because endothelium-dependent relaxation is fundamental to vascular homeostasis, and its impairment represents an early pathological event in hypertension and vascular remodeling (Augustin and Koh, 2024; Förstermann and Münzel, 2006). Although previous studies demonstrated that ClC-3 deficiency attenuates cerebrovascular remodeling in DOCA-salt hypertension primarily through TGF-β–dependent extracellular matrix pathway (Zheng et al., 2013), and independent work has shown that ClC-3 exacerbates endothelial inflammation via NF-κB activation and superoxide generation (Yang et al., 2012; Hawkins et al., 2007), our current findings suggest a more fundamental role of ClC-3 in the maintenance of vascular homeostasis. This multiple benefit of ClC-3 inhibition provides a robust mechanism for the observed vascular protection.

Endothelial dysfunction, characterized by impaired NO bioavailability and reduced endothelium-dependent relaxation, is a central feature of hypertension and significantly contributes to its major cardiovascular and metabolic complications (Baaten et al., 2023; Xu et al., 2021; Cho et al., 2025; Jin et al., 2021; Zhou et al., 2025). Previous studies have established that caveolin-1 binds to and inhibits eNOS catalytic activity (Maniatis et al., 2006; Ju et al., 1997), while Hsp90 acts as a molecular scaffold that facilitates the interaction between Akt and eNOS. Hsp90 enhances eNOS phosphorylation by increasing Akt-eNOS affinity and by exposing critical phosphorylation sites on eNOS (Brouet et al., 2001; Sato et al., 2000).

To define the mechanism by which ClC-3 regulates eNOS activity, we assessed whether ClC-3 influences eNOS association with caveolin-1. Co-immunoprecipitation assays demonstrated ClC-3 directly interacts with eNOS, More importantly, ClC-3 deficiency promoted dissociation of eNOS from caveolin-1, consistent with the relief of vascular tone and enhanced eNOS activation. These data indicate that ClC-3 binding favors an inhibitory eNOS conformation. In addition, ClC-3 deficiency was associated with increased Akt phosphorylation and enhanced formation of the eNOS-Hsp90-Akt complex. Thus, removal of ClC-3 not only relieves caveolin-1-mediated inhibition but also facilitates recruitment of the Hsp90-Akt signaling module necessary for full eNOS activation.

Structurally, eNOS consists of an N-terminal oxygenase domain (NOSox domain) that binds heme, the substrate L-arginine, and the cofactor tetrahydrobiopterin (BH_4_), and a C-terminal reductase domain (NOSre domain) containing NADPH, FAD, and FMN binding sites required for electron transfer (Förstermann and Sessa, 2012). These domains are connected by a calmodulin (CaM) binding region (Förstermann and Sessa, 2012; Raman et al., 1998). Functional eNOS exists as a homodimer stabilized by BH_4_ and Zn^2+^ and catalyzes NO production through electron transfer from NADPH to heme via FAD and FMN. Electron flow from the reductase domain of one monomer to the oxygenase domain of the opposing monomer is initiated by Ca^2+^/CaM-dependent signaling. Domain specific analysis of eNOS therefore provides a useful framework for dissecting its regulation by interacting proteins.

Our experimental results showed that deletion of the eNOS oxygenase domain (1–511 aa) abolished the direct interaction between ClC-3 and eNOS, indicating that the oxygenase domain is essential for ClC-3 binding. ClC-3 may interact specifically with the eNOS oxygenase domain. Previous studies have demonstrated that Hsp90 binds to amino acids 310–323 within the eNOS oxygenase domain (García-Cardeña et al., 1998; Fontana et al., 2002), while Akt also exhibits a weaker interaction with eNOS through its pleckstrin homology (PH) domain (Dimmeler et al., 1999; Fulton et al., 1999). Because ClC-3, Hsp90, and Akt all interact with the eNOS oxygenase domain, it is likely that ClC-3 competes with the Hsp90-Akt complex for eNOS binding. Consequently, loss of ClC-3 facilitates the formation of the Hsp90-Akt-eNOS complex and promotes the dissociation of eNOS from caveolin-1, thereby enhancing eNOS phosphorylation. In addition, increased Akt phosphorylation levels may also contribute, at least in part, to the upregulation of eNOS phosphorylation (the schematic was shown in Figure 6E).

This mechanistic insight is strongly supported by prior evidence that disruption of Hsp90-eNOS binding decreases NO output, and further validated by the established link between chloride conductance and oxidative stress (Liang et al., 2018; García-Cardeña et al., 1998; Pritchard et al., 2001). The concurrent observation that ClC-3 deletion reduces endothelial ROS and NF-κB mediated inflammatory signaling supports a comprehensive vascular protection effect of ClC-3 inhibition (Liang et al., 2018; Yang et al., 2012). With these regulatory functions, ClC-3 emerges as a highly attractive therapeutic target for vascular protection in hypertension. Moreover, given the increasingly recognized link between impaired endothelial eNOS function and systemic insulin resistance, modulating ClC-3 activity could offer a novel strategy to address the dual pathology of hypertension-related diabetes.

In the present study, we focused on the interaction between ClC-3 and eNOS to clarify the mechanism by which ClC-3 regulates endothelial function. However, as a member of the voltage-gated chloride channel family and a Cl^−^/H^+^ exchanger, ClC-3 may also modulate intracellular chloride concentration, which could in turn affect NO production and eNOS activation. In line with this notion, our recently published work demonstrated that reducing intracellular Cl^−^ levels using a low Cl^−^ extracellular solution impairs endothelial NO production (Sun et al., 2025). On the other hand, eNOS uncoupling is recognized as a key mechanism underlying impaired NO bioavailability. Given that our previous studies have shown that ClC-3 mediates AngII-induced NADPH oxidase (NOX) activation and ROS production (Liang et al., 2018; Liu et al., 2013), it is plausible that ClC-3 may also regulate AngII-induced eNOS uncoupling through NOX-derived ROS. In addition, although our findings in HUVECs provide novel mechanistic insight, validation in arterial endothelial cells such as HAECs or HCAECs, as well as endothelial cells derived from different vessels, will further strengthen the translational potential of ClC-3.

Future investigation must prioritize several avenues to advance these findings toward clinical application. We will focus on mutational and structural analyses to precisely map the ClC-3 binding site on the NOSox domain and its competitive interface with Hsp90/Akt, thereby enabling the rational design of selective inhibitors. Furthermore, rigorous evaluations of the chronic efficacy and systemic safety of candidate ClC-3 modulators are imperative, including potential off-target effects on epithelial ion transport and electrolyte balance. Collectively, these data establish ClC-3 as a novel and critical mediator of endothelial dysfunction. Continued mechanistic exploration and translational validation may position ClC-3 as a promising therapeutic target for restoring endothelial function and improving long-term outcomes in patients with vascular disorders.

## Data Availability

The raw data supporting the conclusions of this article will be made available by the authors, without undue reservation.

## Supplementary material

SUPPLEMENTARY FIGURE S1 Blood pressure in AngII induced hypertensive mice. Blood pressure was elevated in both wild-type (WT) and ClC-3 knockout (KO) mice after chronic AngII infusion for 2 weeks (n=6, *P<0.05 vs. con).

SUPPLEMENTARY FIGURE S2 Effect of ClC-3 shRNA on eNOS protein expression and phosphorylation in HUVECs. ClC-3 knockdown in HUVECs using shRNA significantly increased eNOS phosphorylation at Ser1177. **P<0.01 vs. Scramble, n=5. Values were normalized to control.

SUPPLEMENTARY FIGURE S3 Effect of ClC-3 shRNA on ClC-3 protein expression in HUVECs. Cells were treated with the 0, 25, 50,100 MOI adenovirus ClC-3 shRNA for 72 h, then the expression of ClC-3 protein was detected by western blot. Results are mean±SEM, *P<0.01 vs. control, n=5. Values were normalized to control.

SUPPLEMENTARY FIGURE S4 Effect of ClC-3 knockout on eNOS expression in aortas from AngII-infused hypertensive mice. ClC-3 deficiency had no significant effect on total eNOS expression in thoracic arteries during hypertension. n=5.
