# Supplementary figures and images for: ClC-3 mediates angiotensin II-induced endothelial dysfunction by inhibiting Akt-Hsp90-eNOS signaling pathway
Source: Front Pharmacol. 2026 Mar 27;17:1795707. doi: 10.3389/fphar.2026.1795707 (PMC13066174; doi:10.3389/fphar.2026.1795707)

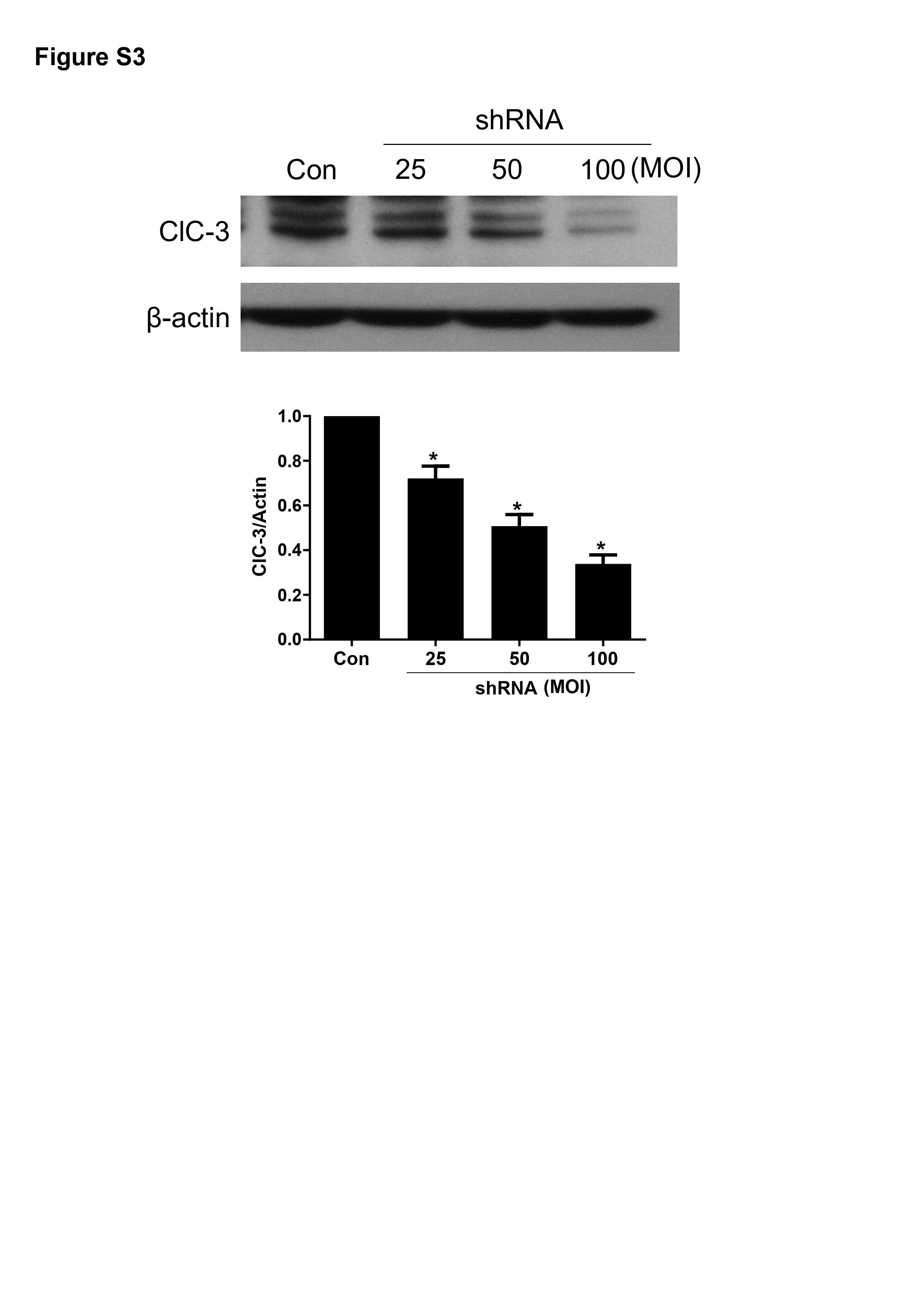

Supplement: Supplementary file 1 [file Image3.jpeg]

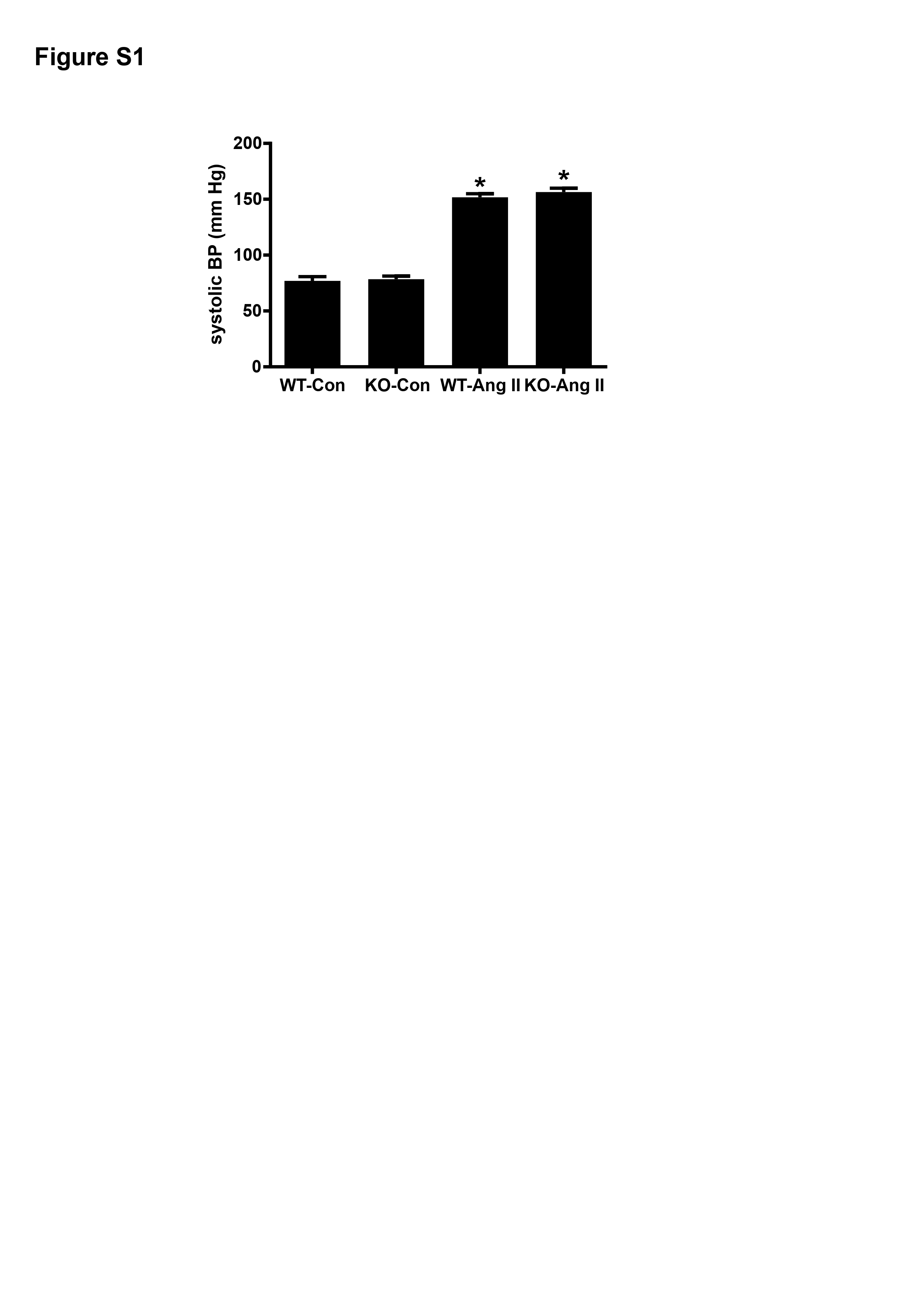

Supplement: Supplementary file 2 [file Image1.jpeg]

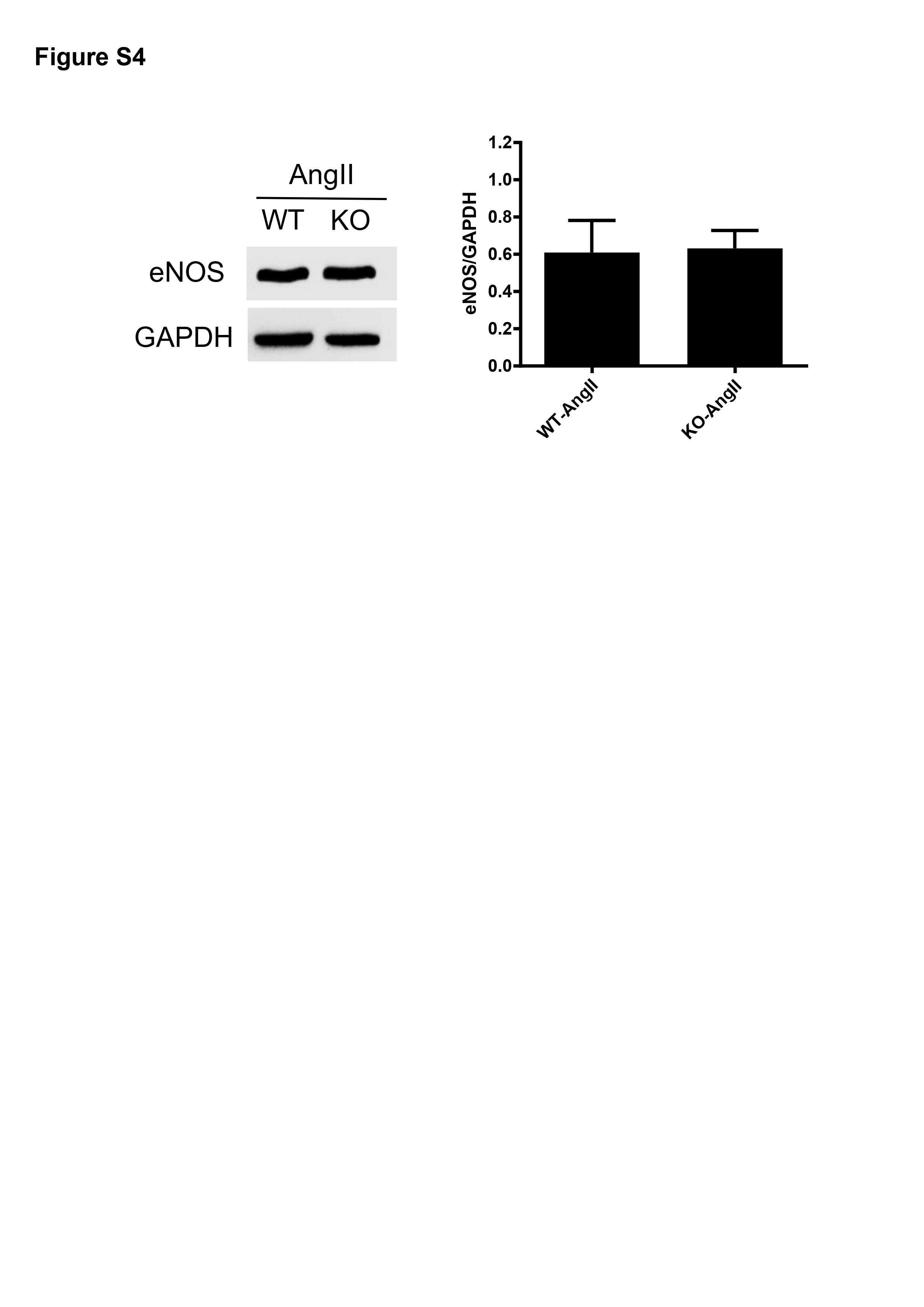

Supplement: Supplementary file 3 [file Image4.jpeg]

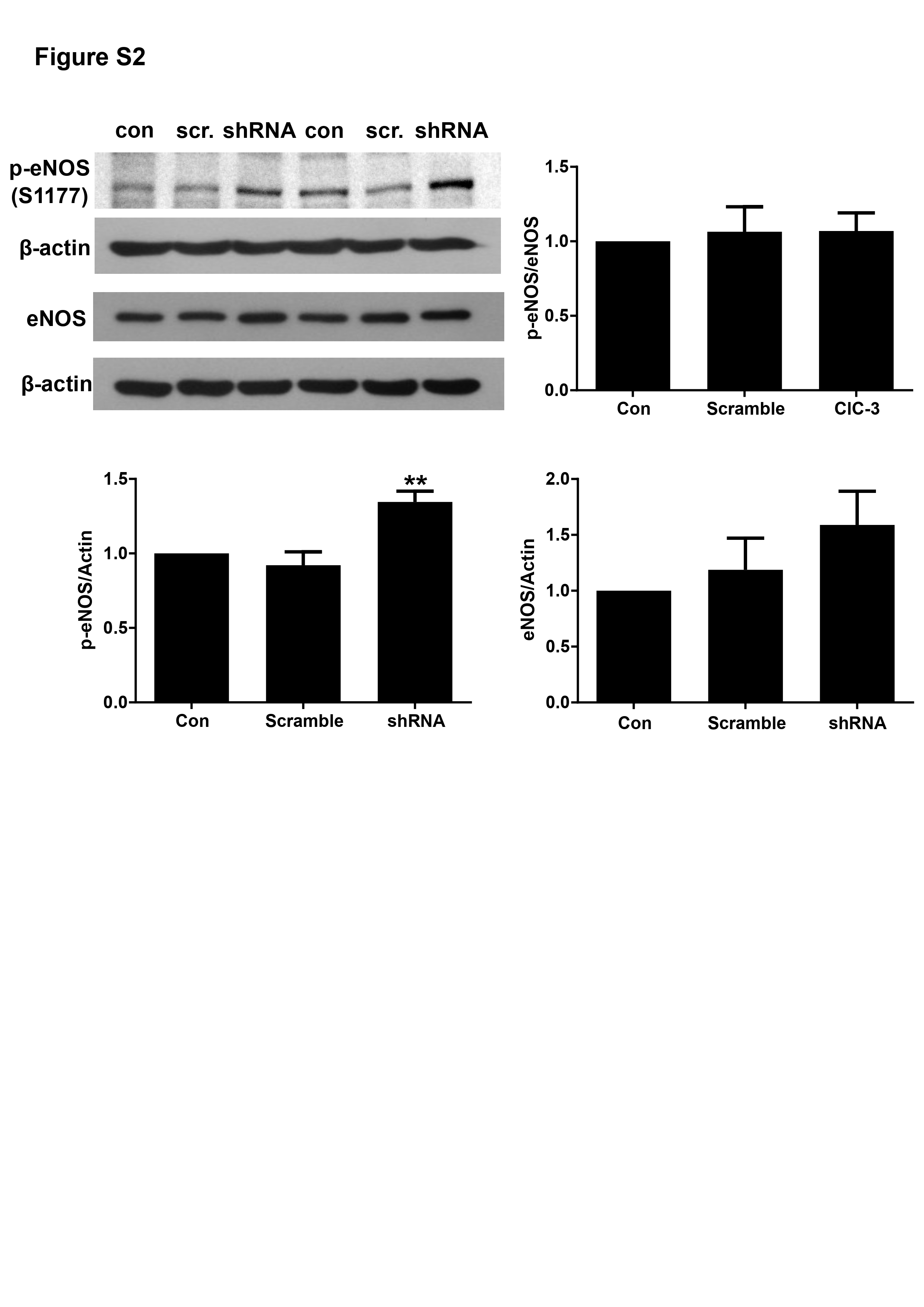

Supplement: Supplementary file 4 [file Image2.jpeg]
